# Multiple sclerosis in Central America and Caribbean countries: frequency and clinical characterization of an emergent disease

**DOI:** 10.3389/fepid.2024.1368675

**Published:** 2024-06-17

**Authors:** Fernando Gracia, Deyanira A. Ramírez Navarro, Nicia E. Ramírez Sánchez, Roberto Weiser, Alexander Parajeles-Vindas, Ligia I. Portillo Rivera, Ericka López Torres, Luis A. García Valle, Alfredo Sanabria-Castro, César Abdón López, Pahola Araujo, Maria J. Ayerdis Zamora, Andrea Balmaceda-Meza, Aron Benzadon Cohen, Awilda Candelario Cueto, Diego Castillo, Romy Castro-Escobar, Karla Z. Corea Urbina, Anyeri de Peña Rivas, Octavio Duarte Sotelo, Temís Enamorado Claros, José L. Giroud Benítez, Karla Gracia, Mario Larreategui, Jorge A. Martínez Cerrato, Josmarlin P. Medina Báez, Carlos E. Menjivar Samayoa, Gustavo Miranda-Loria, Priscilla Monterrey-Alvarez, Lilliam A. Morales Arguello, Michelle Ortiz, Carlos D. Pérez Baldioceda, Lizeth Pinilla Aguilar, Luis C. Rodríguez Salinas, Virginia Rodríguez-Moreno, Sebastián Rojas-Chaves, Norbel Román-Garita, Biany Santos Pujols, Carlos Valderrama, Ivonne Van Sijtveld, Indhira Zabala Angeles, Victor M. Rivera, Blas Armien

**Affiliations:** ^1^ Neurology Service, Hospital Santo Tomás, Panamá, Panama; ^2^ Dean of Health Science Faculty, Universidad Interamericana de Panamá, Panamá, Panama; ^3^ Neurology Service, Hospital Docente Padre Billini, Santo Domingo, Dominican Republic; ^4^ Neurology Service, Hospital Público Dr. Mario C. Rivas, San Pedro Sula, Honduras; ^5^ Neurology Service, Hospital Horacio Oduber, Oranjestad, Aruba; ^6^ Neurology Service, Hospital San Juan de Dios, Caja Costarricense de Seguro Social (CCSS), San Jose, Costa Rica; ^7^ Neurology Service, Hospital General de Enfermedades, Instituto Guatemalteco de Seguridad Social, Guatemala, Guatemala; ^8^ Neurology Service, Hospital Nacional Rosales, San Salvador, El Salvador; ^9^ Neurology Service, Hospital Militar Escuela Dr. Alejandro Dávila Bolaños, Managua, Nicaragua; ^10^ Neurology Service, Complejo Hospitalario Dr. Arnulfo Arias Madrid, Panama, Panama; ^11^ Neurology Service, Hospital Bautista de Nicaragua, Managua, Nicaragua; ^12^ Neurology Service, Instituto Salvadoreño del Seguro Social, San Salvador, El Salvador; ^13^ Neurology Service, Centro de Diagnóstico, Medicina Avanzada Conferencias Médicas, Telemedicina (CEDIMAT), Santo Domingo, Dominican Republic; ^14^ Neurology Service, Instituto Hondureño de Seguro Social, Tegucigalpa, Honduras; ^15^ Neurology Service, Hospital Salud Integral, Managua, Nicaragua; ^16^ Neurology Service, Hospital Regional Anita Moreno, Villa de Los Santos, Panama; ^17^ Neurology Service, Hospital Vivian Pellas, Managua, Nicaragua; ^18^ Neurology Service, Hospital San Rafael de Alajuela, Caja Costarricense de Seguro Social (CCSS), Alajuela, Costa Rica; ^19^ Neurology Service, Hospital San Carlos, Caja Costarricense de Seguro Social (CCSS), San Carlos, Costa Rica; ^20^ Neurology Service, Hospital Regional Universitario Jose Maria Cabral y Baez, Santiago De Los Caballeros, Dominican Republic; ^21^ Neurology Service, Hospital Regional Rafael Hernández, David, Panama; ^22^ Neurology Service, Baylor College of Medicine, Houston, TX, United States; ^23^ Directorate of Research and Technological Development, Gorgas Memorial Institute of Health Studies, Panamá, Panama; ^24^ Sistema Nacional de Investigación, Secretaria Nacional de Ciencia y Tecnología, Panamá, Panama

**Keywords:** multiple sclerosis, incidence, prevalence, Mestizos, Central America, Caribbean

## Abstract

**Background:**

Multiple Sclerosis (MS) is a common neurological disease among white populations of European origin. Frequencies among Latin Americans continue to be studied, however, epidemiologic, and clinical characterization studies lack from Central American and Caribbean countries. Ethnicity in these countries is uniformly similar with a prevalent Mestizo population.

**Methods and results:**

Data from January 2014 to December 2019 from Guatemala, El Salvador, Honduras, Nicaragua, Costa Rica, Panama, Dominican Republic, and Aruba on demographic, clinical, MRI and phenotypic traits were determined in coordinated studies: ENHANCE, a population-based, retrospective, observational study on incidence and clinical characteristics, and from the subgroup with MS national registries (Aruba, Dominican Republic, Honduras, and Panama), data on prevalence, phenotypes and demographics. Expanded Disability Status Scale (EDSS), and therapeutic schemes were included. ENHANCE data from 758 patients disclosed 79.8% of Mestizo ethnicity; 72.4% female; median age at onset 31.0 years and 33.2 at diagnosis. The highest incidence rate was from Aruba, 2.3–3.5 × 100,000 inhabitants, and the lowest, 0.07–0.15 × 100,000, from Honduras. Crude prevalence rates per 100,000 inhabitants fluctuated from 27.3 (Aruba) to 1.0 (Honduras). Relapsing MS accounted for 87.4% of cases; EDSS <3.0 determined in 66.6% (mean disease duration: 9.1 years, SD ± 5.0); CSF oligoclonal bands 85.7%, and 87% of subjects hydroxyvitamin D deficient. Common initial therapies were interferon and fingolimod. Switching from interferon to fingolimod was the most common escalation step. The COVID-19 pandemic affected follow-up aspects of these studies.

**Conclusion:**

This is the first study providing data on frequencies and clinical characteristics from 8 countries from the Central American and Caribbean region, addressing MS as an emergent epidemiologic disorder. More studies from these areas are encouraged.

## 1 Introduction

Multiple Sclerosis (MS), an autoimmune, inflammatory, demyelinating, and neurodegenerative central nervous system disorder, is common in young adults and affects predominantly females. The disease is more common among white Caucasians of Northern European descent and populations in the world sharing that ancestry pool: most of Northern European countries, Canada, Northern United States, Southeastern Australia, and New Zealand, all these regions situated away from the Equator (1). The area with the highest prevalence of MS in the world is the Orkney Island in Northeastern Scotland, reported in 2021 as 402/100,000 inhabitants (2). Most MS epidemiologic studies in the world address prevalence while annual incidence remains undetermined in most places. Epidemiologic studies In Latin American countries have shown divergent prevalence frequencies from 0.76/100,000 in Guayaquil, Ecuador, to 38.2/100,000 inhabitants in Buenos Aires, Argentina (3). The first study from Central America (Panama, 2009), reported a lower prevalence: 5.84/100,000 with an incidence between 0.28 to 0.61/100,000 population (4). Most of the subjects in this report were diagnosed utilizing the Poser's criteria (5).

**Figure 1:**
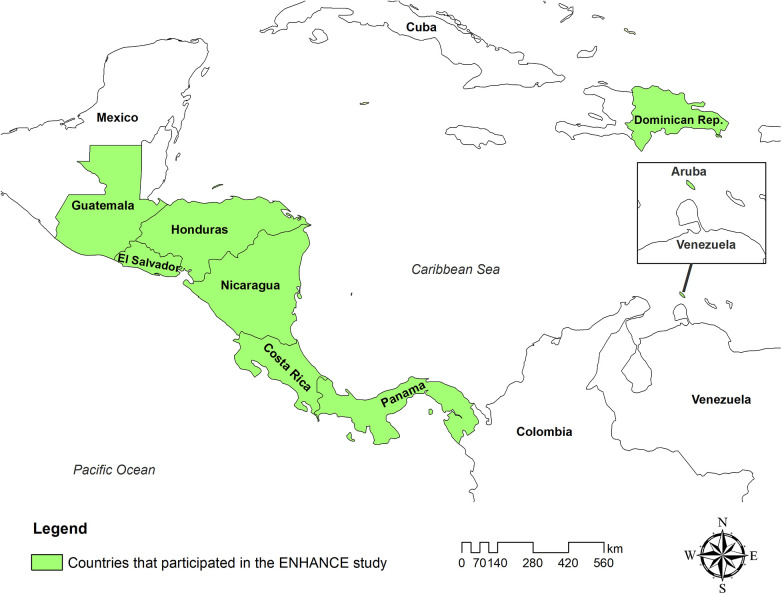
Map of the Central America and Caribbean countries that participated in the ENHANCE study.

Central America is the southernmost part of North America on the Isthmus of Panama, that links the continent to South America. It is constituted by the countries south of Mexico: the territory of Belize, Guatemala, El Salvador, Honduras, Nicaragua, Costa Rica, and Panama. The Caribbean is the region south of the United Sates, east of Mexico and north of Central and South America, consisting of the Caribbean Sea and its islands (Figure 1).

MS clinical manifestations including demographics and clinical phenotypes, degree of neurological disability, Magnetic Resonance Imaging (MRI) characteristics, and long-term prognosis, have not been studied in Central American and Caribbean countries. The racial and ethnic composition among these populations is consistently uniform in the region. The predominant population group, as in most areas of Latin America, is represented by Mestizos. It is generally considered the main genetic risk factor predisposing to MS in the Americas (6). This historic genetic intermixing (European/Indigenous people of the Americas) took place over the course of five centuries in the American continent. In the present study, an exception to the traditional Hispanic mestizaje recognized for Central America and the Caribbean, it is represented by the inclusion of the country island of Aruba, which carries a more complex racial admixture. Over the course of time, interest in MS epidemiology in Latin America (LATAM) has evolved (7, 8), however, information from Central American and Caribbean countries remains scanty.

As an emergent disease in a region where considerable economic limitations exist, MS annual incidence, the clinical characterization of the disorder and its phenotyping, are factors of interest considering the potential societal impact, and the effect in patient perspectives, and in health-related quality of life aspects (9). The advent of Magnetic Resonance Imaging (MRI) in the diverse areas of LATAM, albeit sub optimally available, has helped to improve the clinical diagnosis of MS efficiently the diverse versions of the McDonald criteria (10–13). Information derived from epidemiologic and clinical characterization studies will be helpful providing data for regional MS surveillance. This is the first work addressing these issues in the context of the MS presence in Central America and the Caribbean.

## 2 Methods and study design

### 2.1 Study group

The ENHANCE study (Incid**EN**ce and C**HA**racteristics of MS patie**N**ts: An Epidemiologi**C**al Study of Multipl**E** Sclerosis in Central America and Caribbean Countries) was designed as a population-based retrospective, regional, observational incidence study conducted to determine all new cases of MS diagnosed between January 2014 and December 2019 in this region. The collection of data was carried out between September 2021 and November 2022. The COVID-19 pandemic interrupted the flow of the study for two years. Medical centers from Guatemala, El Salvador, Honduras, Nicaragua, Costa Rica, Panama, Dominican Republic, and Aruba, contributed to the study. To allow comparability between these countries' population as a region, data from the United Nations Economic Commission for Latin America and the Caribbean (ECLAC) -or CEPAL Spanish acronym-, were utilized. Populations from the most recent national census were used as the standard population for the frequency estimates (14), verified with the national census institutes from the countries participating in the study.

**Figure 2:**
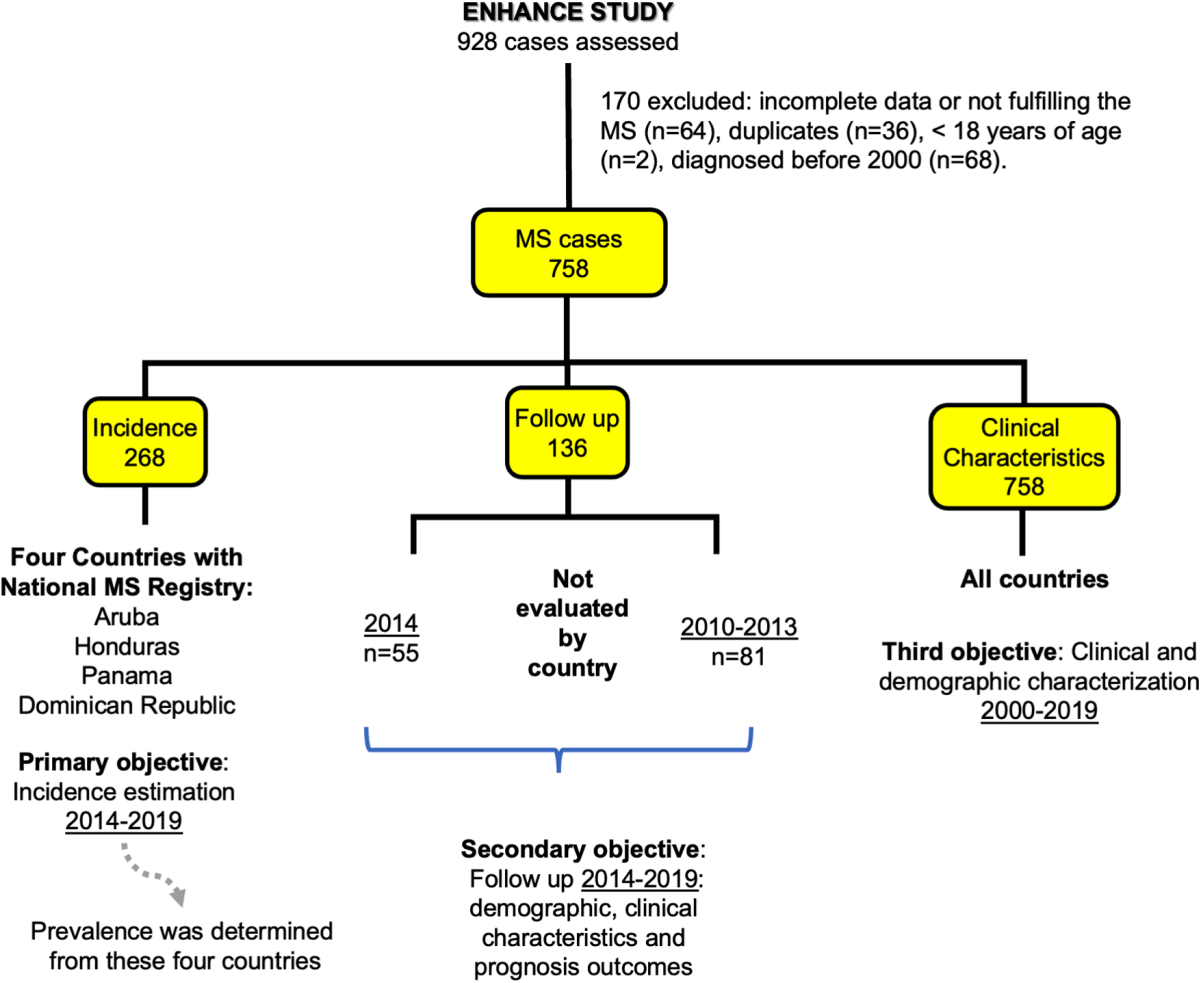
Flowchart of MS patients included in the ENHANCE study.

In view that some participating countries lacked a national MS registry, patients were included from January 2000 and December 2019 and identified according to the inclusion criteria: new patients older than 18 years of age, diagnosed with MS applying the different versions of the McDonald criteria, according to the epoch studied. Patient data were gathered from the medical files of contributing centers. Data on demographics (age, sex, and ethnicity), age at onset, time to diagnosis, MS type, and Expanded Disability Status Scale (EDSS) were collected from this core group. EDSS reported was the last recorded in the clinical chart. The same sources and national repositories were employed to study prevalence from 2000 to 2019 (Figure 2).

To study incidence, data from 2014 to 2019 acquired from the national MS registries from a subgroup formed by Aruba, Dominican Republic, Honduras, and Panama, were utilized (*n* = 268). Other subgroups to assess specific features arising during a three-year follow-up from January 2010 and December 2013, were included from patients that fulfill inclusion criteria, and another set of subjects was extracted from the 2014 incidence group. The patients constituting these cohorts were followed retrospectively and had at least one annual follow-up during the period studied from January 2014 to December 2019 (Figure 2). In addition to the demographic information and EDSS scores, data on brain and spinal cord MR imaging, optic coherent tomography (OCT), presence of oligoclonal bands in Cerebrospinal fluid (CSF), and serum hydroxyvitamin D levels, were included. Prescribed treatments employed were also analyzed.

### 2.2 Data collection

All the information was collected separately from different centers and transferred to a secure electronic database at the Gorgas Memorial Institute for Health Studies (GMIHS) in Panama City, Panama. This was compiled in a single repository. To assure validity and quality of data, two separated controls were carried out: by the GMIHS and by an independent external group.

### 2.3 Statistical analysis

Counts and percentages were provided for categorical variables, and continuous variables were expressed as mean (with standard deviation) or median with inter-quarter range (IQR). Calculations of crude incidence rate with 95% confidence interval differences were used in the studied population utilizing normal standard deviation. The crude annual incidence rate was estimated using the number of new MS cases during the year among the population estimated at risk as of July 1 of the respective year and the corresponding geographic area or country. The crude prevalence rate was estimated using the total number of MS in a population divided by the estimated total population as of July 1, 2019, corresponding to the geographic area or country. The statistical software for epidemiology Epi Info (version 7.2.4. 27 April 2020) and Microsoft® Excel® 2013 (version 16/73), were utilized. The information was available online allowing data collection to be followed remotely by the study investigators from each country, ensuring the confidentiality of the data. All researchers participating in this study had access to their data, and the gathered information which, eventually, will be available to all interested professionals from the region.

### 2.4 Ethical considerations

A consent form was not applicable as ethical review and approval were not required for the study on human participants in accordance with the existing local legislation and institutional requirements. All personal information was removed to perform the analysis and patient identification was codified to respect confidentiality, complying with international human research protections policy and exemptions.

## 3 Results

The ENHANCE study collected data from the eight contributing countries for the study period between January 2000 and December 2019. The data was provided by 43 neurologists from 22 health MS centers, 93.5% belonging to the public health sector. The initial registration of 928 patients was adjusted to 758 cases for study analysis (Figure 2), after assessing patients for eligibility discarding duplicate cases (3.9%), the ones not fulfilling the MS diagnostic criteria, and subjects younger than 18 years. The distribution of cases from the final group were from Dominican Republic 220 (29.0%), Panama 189 (24.9%), Costa Rica 134 (17.7%), Honduras 95 (12.5%), Guatemala 47 (6.2%), Nicaragua 28 (3.7%), Aruba 24 (3.2%), and El Salvador 21 (2.8%). Women accounted for 72.4% of patients with the highest proportion recorded from Panama: 80.4%. The Mestizo ethnicity was identified in 79.8% of these multinational cohorts. Other variables included median age (IQR) at onset 31.0 years (23.6–41.2), and 33.2 (25.6–43.1) at diagnosis. The median time to diagnosis was 6.8 months (2.3–28.5) (Table 1). The different versions of the McDonald criteria for diagnosis of MS were applied by the investigators during the period studied by ENHANCE. Utilization varied among the participating countries.

**Table 1:** Demographic characteristics of the MS population from different countries of Central America and the Caribbean in the years 2000–2019.

Country	*n*	Gender	Mestizo ethnicity (%)	Median age at onset^a^			Median age at diagnosis^b^			Median time to diagnosis (Months)^c^		
		(% F)		Median age	IQR^d^	IQR	Median age	IQR	IQR	Median time	IQR	IQR
Aruba	24	66.7	79.2	38.1	26.8	47.9	38.4	27.1	49.0	5.0	3.7	5.5
Costa Rica	134	68.7	–^e^	30.9	24.6	40.3	33.5	27.1	43.1	12.2	3.4	37.4
El Salvador	21	76.2	100.0	25.6	19.5	30.6	27.5	20.5	33.1	12.1	3.3	27.6
Guatemala	47	70.2	93.6	29.7	26.0	38.9	30.8	26.7	40.1	6.4	3.3	16.8
Honduras	95	67.4	90.5	28.3	20.9	40.1	30.0	23.7	42.4	7.2	2.1	30.4
Nicaragua	28	75.0	96.4	28.1	20.9	39.9	30.8	24.0	40.1	8.1	1.3	28.1
Panamá	189	80.4	64.0	36.0	27.7	44.1	38.3	29.6	44.9	5.7	2.1	18.7
Dominican R.	220	70.5	81.8	29.5	22.7	37.9	31.6	24.6	39.8	7.9	2.1	34.5
Total	758	72.4	79.8	31.0	23.6	41.2	33.2	25.6	43.1	6.8	2.3	28.5

^a^ Median age at onset: patient's age at first symptom.

^b^ Median age at diagnosis: age when diagnosis was confirmed per neurologist.

^c^ Median time to diagnosis (months): was estimated by subtracting the date the diagnosis was confirmed from the date the first symptom occurred.

^d^ Lower and upper bound Interquartile Range (IQR).

^e^ Information not available in the country database.

**Figure 3:**
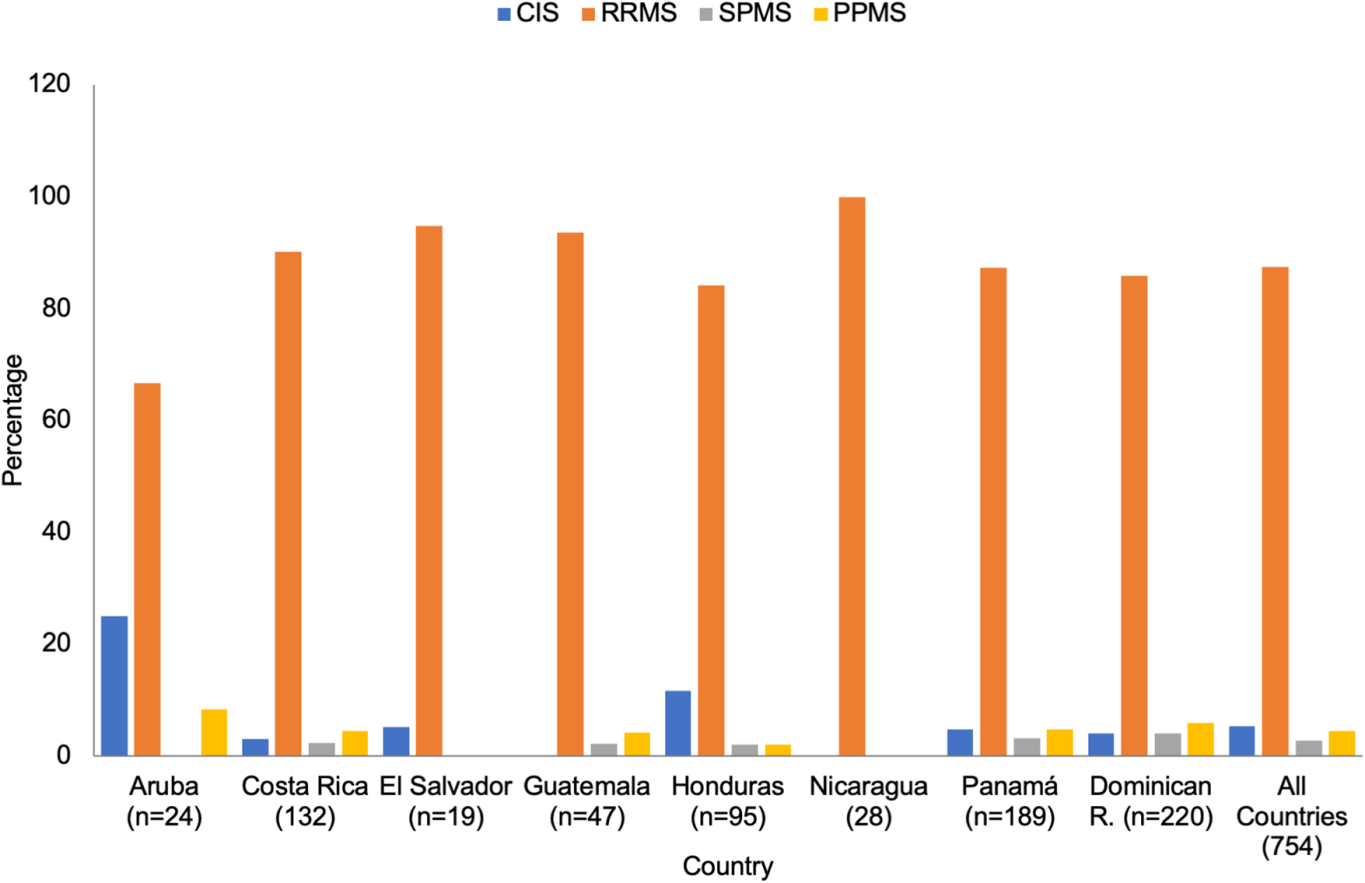
Type of MS by country (*n* = 758). CIS, clinically isolated syndrome; RRMS, relapsing remitting MS; SPMS, secondary progressive MS; PPMS, primary progressive MS.

The most common MS clinical form reported was a relapsing/remitting course (RRMS), accounting for 87.4% (varying from 66.7% in Aruba to 100% in Nicaragua) of the cases. Clinically isolated syndrome (CIS) was reported in 5.3% (0.0%–25.0%) of the cohorts; the primary progressive form (PPMS) in 4.5% (0.0%–8.3%), and a secondary progressive form (SPMS) in 2.8% (0.0%–4.1%) of all cases (Figure 3).

**Figure 4:**
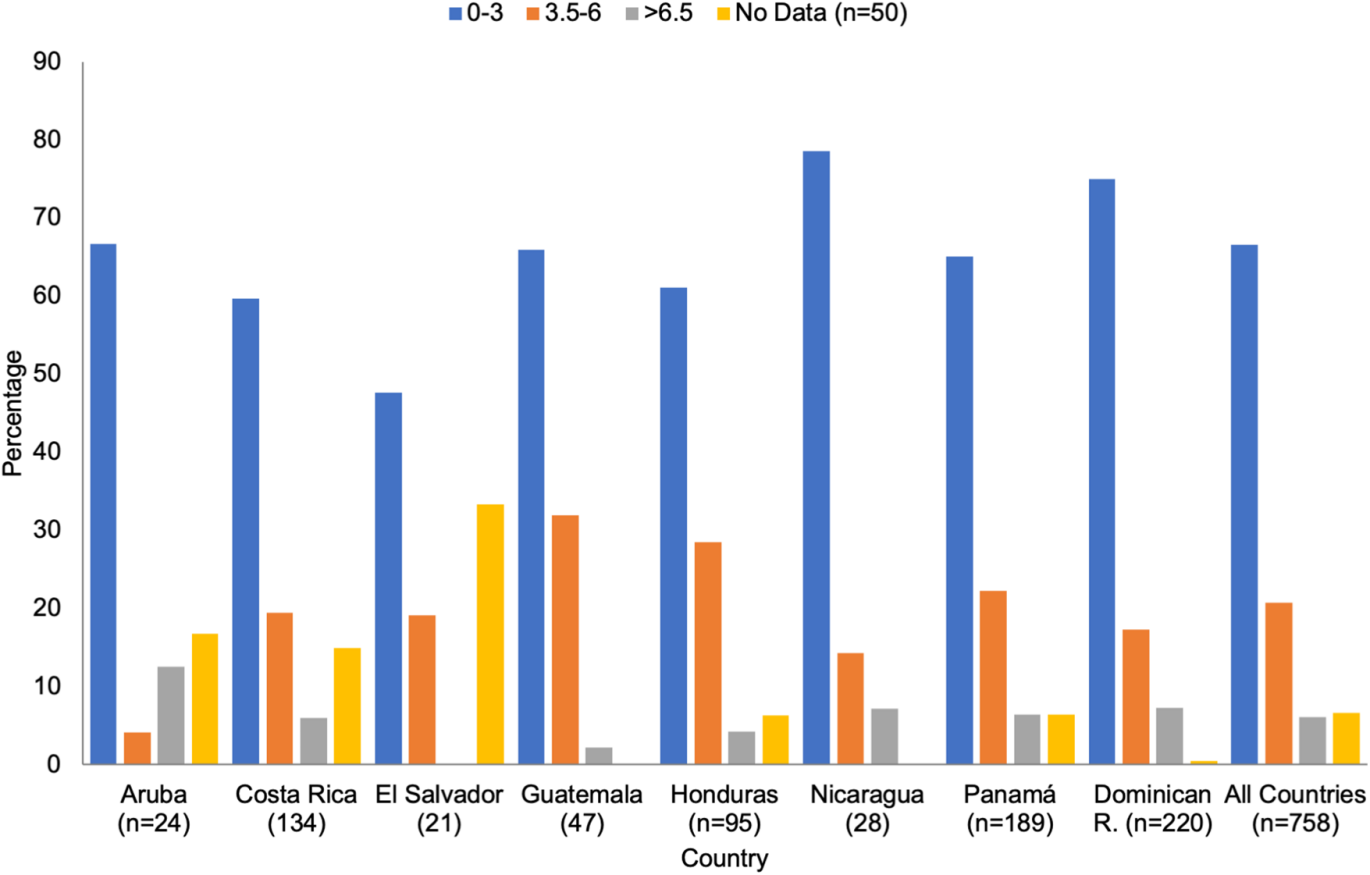
Expanded disability Status scale by country.

Neurological disability was estimated utilizing the EDSS scores. The largest proportion of individuals (66.6%: varying from 47.6% in El Salvador to 78.6% in Nicaragua; mean disease duration: 9.1 years, SD ± 5.0) were adjudicated scores in the range of 0.0 to 3.0. High disability rates (>6.5) were reported in 6.1% of cases (mean disease duration: 13.1 years, SD ± 5.7), the higher proportion occurring in Aruba (12.5%), and the lowest reported from El Salvador (0.0%) (Figure 4).

MRI data was available from 701 patients. Location of lesions was described as affecting periventricular areas (88.2%), juxtacortical/cortical regions (60.2%), spinal cord (45.9%), and infratentorial structures (44.9%). Information was not retrievable from 57 individuals.

**Figure 5:**
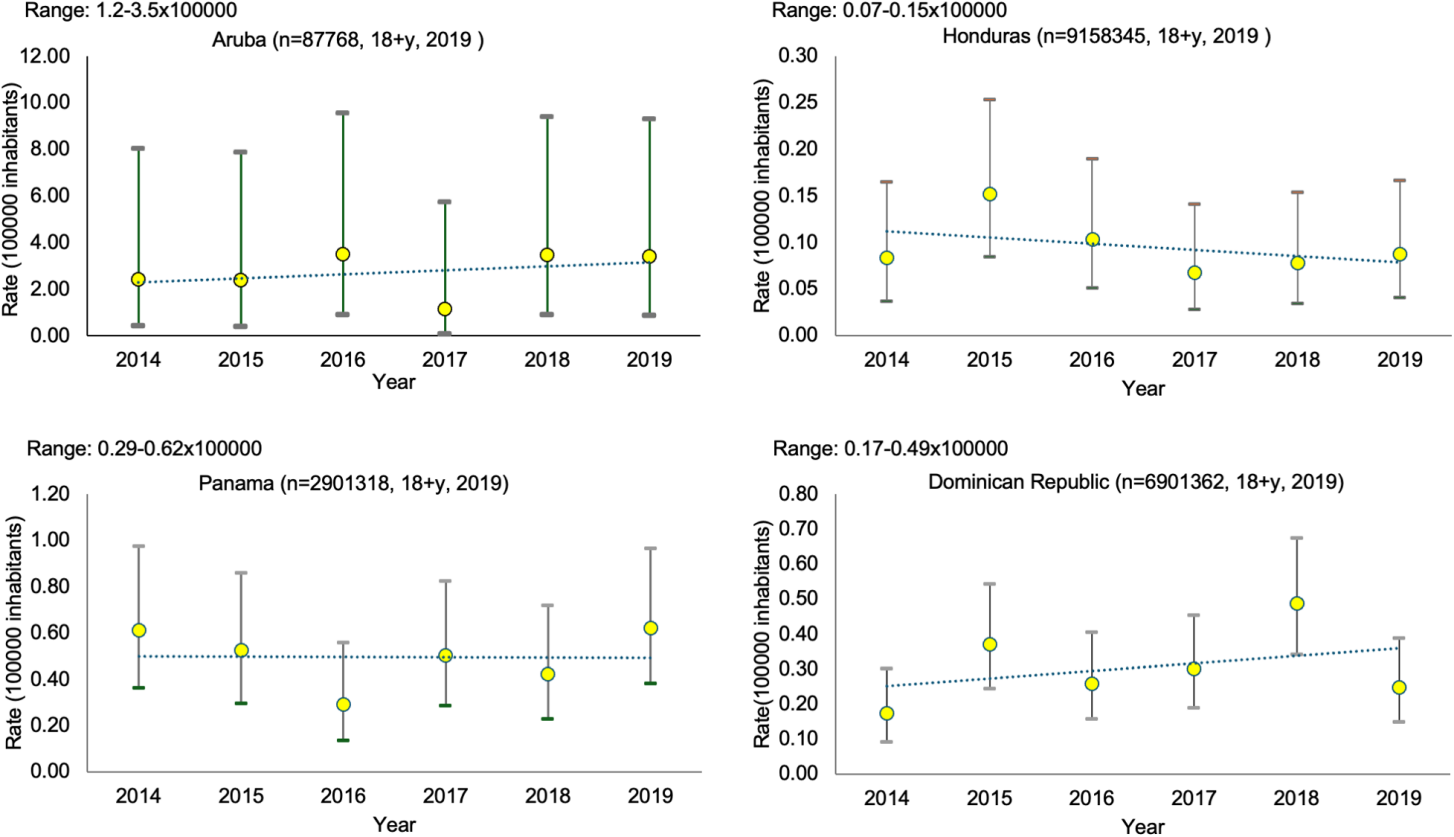
Crude incidence rate of multiple sclerosis by country, 2014–2019. Range, crude incidence rate; UCI, upper confidence interval and LCI, lower confidence interval.

Crude incidence rate was determined retrospectively from 2014 to 2019, from the only four countries that counted with national MS registries two Central American countries: Honduras and Panama, and two Caribbean countries: Aruba and Dominican Republic, compiled a total of 268 individuals. The highest rate, 2.4–3.5 × 100,000 inhabitants, was recorded in Aruba, and the lowest, 0.07–0.15 × 100,000 inhabitants, in Honduras. During this time, incidence rates in Panama were 0.29–0.62 × 100,000 and 0.17–0.49 × 100,000 in Dominican Republic (Figure 5). Crude prevalence rates per 100,000 inhabitants in these countries showed variations: Aruba 27.3, Panama 6.5, Dominican Republic 3.2, and Honduras 1.0 (Table 2). In the subgroup utilized to study specific features (*n* = 136) (Figure 2), CSF analysis exhibited oligoclonal bands on 85.7% (48/56) of the patients. OCT was performed in 41 patients demonstrating optic nerve pathology in 23 individuals. Serum hydroxyvitamin D levels were measured in 53 patients: 28 were insufficient with ranges between 21.0 ng/ml–39.9 ng/ml, and 18 were deficient with levels of ≤20.0 ng/ml.

**Table 2:** Crude prevalence rate of multiple sclerosis from different countries of Central America and the Caribbean in the years 2000–2019^a^.

Country	Cases of MS	Population^b^ (18+ years old)	Rate (10^5^ inhabitants)
Aruba	24	87,768	27.3
Honduras	95	9,158,345	1.0
Panama	189	2,901,318	6.5
Dominican Republic	220	6,901,362	3.2

^a^ All patients with MS were identified from the national medical records for the period January/01/2000 to December/31/2019. McDonald Criteria 2000, 2005, 2010 and 2017 were applied accordingly.

^b^ Population estimated July 1st, 2019.

Utilization of disease modifying therapies (DMT) identified in the follow up (Figure 2) showed the most common initial agent was beta interferon (105/136; 77.2%), followed by fingolimod (15/136; 11.0%); but also, the most frequent DMT switch was from beta interferon to fingolimod (40/136; 29.4%). Escalation to another therapy took place in 39.7% (54/136) of patients while utilization of a third medication, comprised of the monoclonal antibodies natalizumab, ocrelizumab and alemtuzumab, occurred in 7.4% (10/136). Other medications: glatiramer acetate, teriflunomide, dimethyl fumarate and cladribine, were used in <5.0%. Patients not receiving treatment accounted for 5.8% of cases.

## 4 Discussion

Epidemiologic studies from LATAM indicate increasing identification of MS in the region. Studies over the course of the last three decades (15, 16), include reports from Mexico and several from South American countries (17–20). These studies show variable zonal frequencies, without a uniform tendency in prevalence.

The highest prevalence rate noted in Aruba with 27.3/100,000 inhabitants, and the lowest in Honduras with 1.0/100,000 inhabitants. This discrepancy may be related to national population density and other factors including socioeconomic barriers in access to diagnostic technology and care of MS across the area. The “Factbook” of the US Central Intelligence Agency (CIA) (21) reports a national Honduran population of 10.28 million and a GDP per capita of $7,162 in 2022. Honduras has a geographical area of 112,492 km². Private practice of medicine is minimal in Honduras while most of the health care is provided by the Ministry of Public Health, and the Honduran Institute of Social Security. Honduras is the second poorest country after Nicaragua in Central America. The same source reports a national population of Aruba in 2021 of 120,917 with a GDP per capita of $51,352. Health care centres in this country island provide services to the public sector and have a high utilization of private health insurance coverage by third parties. Aruba has a geographical area of 193 km². Even though Aruba is a developing country, per capita income is one of the highest in the Caribbean region. Aruba's genetic composition is constituted by a complex European/Latin American admixture, with a strong Dutch ancestry component. Each country participating in this study faces different but substantial economic concerns, and health access challenges.

Few incidence studies have been carried out in LATAM reporting incidence. Studies from the country of Panama reported an incidence of 0.28–0.61/100,000 for the period 2000–2005. Over the last decade, MS incidence determinations have also been performed in the city of Buenos Aires, Argentina (0.6/100,000), and the countries of Chile (1.81/100,000) and Uruguay (2.24/100,000) (22–24). The incidence rates in the countries comprising our study fluctuated or remained stable but did not increase during the study period. The median age of onset was 31.0. This finding differs from other studies reporting a younger age of onset of disease in Latin American populations in the US: 28.4 ± 0.97 (25). This study compared Latin American groups with MS (Mexicans, Cubans, Puerto Ricans, Central and South Americans) born in Los Angeles (LA) County, California, and Mexicans with MS born in Mexico migrating to LA after age 15. This latter group showed a mean of 34.0 ± 2.31 years of age at first symptom of disease (*p-*value 0.0001). The authors propose as possible explanations for earlier age of diagnosis for Latin American groups born in LA, better access to health care and different environmental exposure. The observed later age in the late migrants may represent restrictions to health care access in the country of origin as well as in the current country (25).

The advent of MRI technology to regions of the world where the presence of MS was considered low in frequency has contributed to increasing identification of the disease. All patients included in the study were required to be diagnosed with MS adhering to the requirements of the criteria in course. MRI findings were in consonance with the typical locations and characteristics of inflammatory demyelinating disease adjudicated to MS (26). Most lesions were localized in periventricular areas (88.2%).

While the relapsing/remitting form of MS was the most frequent clinical type reported (87.4%), the primary progressive form was more common than the secondary progressive type (3.0%–5.0% vs. <5.0%). These results were extracted from the data provided by the 22 health MS centers contributing to this study. Future studies will help to elucidate the factors and reasons affecting this unexpected clinical distribution of progressive disease (Figure 3). Mild disability of 0.0–3.0, as measured by the EDSS, was reported in 66.6%. Higher disability, >6.5 affected 2.0% to 11.0% of the MS population in the region (Figure 4).

In the follow-up subgroup study (*n* = 136), oligoclonal bands in CSF were determined in 56 patients, 85.7% resulting positive, conforming with the traditionally reported proportion of this immunologic abnormality in MS.

Serum hydroxyvitamin D determined in 53 individuals showed 87% to be either insufficient or deficient. This finding is of particular interest considering the Central American and Caribbean regions are tropical areas located just under the Tropic of Cancer, between 7° North and 20° North latitude. The World Health Organization (WHO) reports a Ultraviolet Radiation Index (URI) with a “high” (6.0–7.0) average annual solar ultraviolet irradiation in these areas (27). This phenomenon: low vitamin D despite high solar irradiation, is also reported in other areas of Latin America and the world. Studies performed in the country of Ecuador, the WHO reports in the capital city of Quito (latitude 0.180653) a URI of “moderate” (3.0–5.0) to “very high” (8.0–10.0) in 40%–76% of the days of the year. A comparative study in Ecuador showed 42% of patients with MS were deficient to vitamin D, while 46% of control, “healthy” individuals showed also deficient levels (28). Interestingly, a large proportion (64.58% to 70.0%) of the general Ecuadorian population has been reported deficient in serum hydroxyvitamin D despite the elevated URI levels present around the year in this zone (29). The MS prevalence in this country is low: 3.88 per 100,000 inhabitants (30). In other areas of the world with a high URI, i.e., Australia (25.27° South latitude), about a quarter of the general population are vitamin D deficient, and the MS prevalence is 131.1 per 100,000 inhabitants (31). Ultraviolet B (UVB) radiation exposure through sunlight has been recognized as the most important source of vitamin D. hence, peoples living in areas of the world, above −33° North latitude and below∼33° South latitude, are naturally deficient. These areas are reportedly the ones with the higher frequencies of MS. Numerous observational studies show a causal relationship between MS and low vitamin D level (32), however, the presence of low vitamin D levels despite high URI, appears to be a common occurrence in several areas of Latin America. In the Australian case, the areas recording low vitamin D levels despite high URI, the contributing factors to this phenomenon have been identified as aged population, darkened skin individuals, veiled women, and low vitamin D intake (33). The potential factors involved in the Latin American case have not been identified.

Countries in the Central American and Caribbean experience an insufficient proportion of neurologists: <1.0 per 100,000 population (34). The WHO considers an appropriate rate of 5.0 neurologists per 100,000 inhabitants; however, this is achievable only in high income countries (35). There is a serious lack of neurology specialists in Latin America, due in large part to very limited institutional opportunities of training throughout the region. Only 0.67% of the residency vacancies in Argentina (2023) were assigned to neurology (36). For many Latin American professionals seeking training in neurology, the only option is to accomplish this abroad. Specialization in neuroimmunology is even more complicated.

Availability of MRI equipment remains suboptimal in the region. Data indicate Japan, US, Western Europe countries and South Korea have >15 MRI units per million population. In Latin America, Mexico reports 2.65 units per million inhabitants, but the rest of the countries, including the Central America and Caribbean area report <0.25 units per million people (37). The slow but gradual availability of MRI facilities most likely has improved the diagnosis efficiency of MS in this region. Access to MS therapy in Latin America has been a pervasive concern since the advent of DMT in 1993. This shortcoming is basically due to socioeconomic factors affecting these countries. Only a few agents from the currently approved therapeutic armamentarium are available in some countries, and their accessibility is not uniform in this region. This limitation impacts not just options to therapy but also realistic deliverance of comprehensive MS management. Insufficient access to MS therapy in Latin America is considered an unmet need, which is being addressed by the different stakeholders: health officials, educational institutions, specific study groups, derived from the Latin American Committee for Treatment and Research in MS (LACTRIMS), and more recently from the Central American and Caribbean Committee for Treatment and Research in MS (CACTRIMS), and patients support groups (38).

### 4.1 Limitations

The investigators acknowledge several limitations in this study. Underreporting may be present including effective case-capture and data entry obstacles in some cases. A continuing effort improving and maintaining the process of accurate data acquisition is already taking place in the institutions participating in this, and projected future multinational studies addressing the frequencies and clinical characterization of demyelinating disorders in the Central American and Caribbean region. Since projections to extend the study and obtain subsequent complementary observations were truncated by the COVID-19 pandemic, some aspects remained to be clarified or followed, including factors influencing the distribution of progressive disease (a higher proportion of primary progressive cases comparison to secondary progressive cases). Also, some of the epidemiologic findings produced by the study, such vitamin D deficiency detected in these cohorts despite residing in the tropics with high exposure to UVB. The inclusion of Aruba, while followed geographic reasons, produced important differences in some outcomes. Nevertheless, the data collected in this study constitutes a firm base for the core investigators to project further explorations into the diverse epidemiologic and clinical aspects revealed by this work. The development in 2018 of the MS Central American and Caribbean Forum (Foro Centroamericano y del Caribe de Esclerosis Múltiple-FOCEM) (39) has improved the quality of national studies and promote collaborative investigations among the countries of the region. FOCEM is a registered non-profit organization headquartered in Panama, constituted by seven neurological associations and 14 patients' societies from nine countries. The research group contributing to this work are FOCEM members and contributed to the development of CACTRIMS.

### 4.2 Conclusions

This is the first multinational study evaluating incidence, prevalence, and clinical aspects of MS in the Central American and Caribbean region. Prevalence and incidence rates are low, exhibiting variations among countries, with intermittency in the annual rates, and no apparent sustained increase in incidence over the course of five years, the duration of the study. Age and sex distribution were similar to those reported in other series. Studies addressing diverse aspects of MS as an emergent disease in the different regions of the Latin American region should be encouraged.

## Data Availability

The raw data supporting the conclusions of this article will be made available by the authors, without undue reservation.
